# A novel approach to terminate roof-dependent atrial flutter with epicardial conduction through septopulmonary bundle

**DOI:** 10.1186/s12872-024-03941-9

**Published:** 2024-07-05

**Authors:** Dongchen Zhou, Biqi Zhang, Cong Zeng, Xiang Yin, Xiaogang Guo

**Affiliations:** Department of Cardiology, The First Affiliated Hospital, Zhejiang University School of Medicine, No. 79 Qingchun Road, Shangcheng District, Hangzhou City, Zhejiang Province 310002 China

**Keywords:** Atrial flutter, Septopulmonary bundle, Catheter ablation, Roof-dependent atrial flutter

## Abstract

Atrial flutter, a prevalent cardiac arrhythmia, is primarily characterized by reentrant circuits in the right atrium. However, atypical forms of atrial flutter present distinct challenges in terms of diagnosis and treatment. In this study, we examine three noteworthy clinical cases of atypical atrial flutter, which offer compelling evidence indicating the implication of the lesser-known Septopulmonary Bundle (SPB). This inference is based on the identification of distinct electrocardiographic patterns observed in these patients and their favorable response to catheter ablation, which is a standard treatment for atrial flutter. Remarkably, in each case, targeted ablation at the anterior portion of the left atrial roof effectively terminated the arrhythmia, thus providing further support for the hypothesis of SPB involvement. These insightful observations shed light on the potential significance of the SPB in the etiology of atypical atrial flutter and introduce a promising therapeutic target. We anticipate that this paper will stimulate further exploration into the role of the SPB in atrial flutter and pave the way for the development of targeted ablation strategies.

## Introduction

Atrial flutter (AFL) is a cardiac arrhythmia characterized by rapid and regular atrial contractions, typically caused by reentrant electrical circuits within the atria [1]. AFL can be classified into two types: typical AFL and atypical atrial flutter [2]. Typical AFL is the most common type, with the reentry circuit typically located in the right atrium, along the cavotricuspid isthmus. On the other hand, atypical AFL refers to flutter types where the reentry circuit is located in the left atrium or other non-typical locations [3]. The electrocardiographic presentation of atypical AFL can exhibit significant variability, potentially showing irregular atrial contraction waveforms [4].

The treatment approach for atypical AFL is like that of typical atrial flutter, involving medication, electrical cardioversion, or ablation procedures, depending on the individual patient’s specific circumstances [5]. Ablation is an interventional treatment method that aims to disrupt the abnormal electrical circuits within the atria to treat atypical atrial flutter [6]. Critical technical facets of ablation encompass precise identification of the abnormal electrical circuit’s location, meticulous regulation of energy source application in terms of timing and intensity, and guaranteeing the safety and efficacy of the ablation procedure. With the increasing number of cases involving radiofrequency ablation for atrial fibrillation (AF), atypical flutter cases are also becoming more prevalent. During atypical episodes, top-dependent AFL is commonly observed. Typically, roof line ablation is performed to block macro-reentry. However, sometimes, despite a clear diagnosis and meticulous ablation, tachycardia persists. In such cases, it is necessary to reassess the initial diagnosis, consider alternative circuits, and reevaluate anatomical structures. This article presents three cases of persistent top-dependent AFL following endocardial posterior wall isolation. We consider the involvement of the SPB in these cases, suggesting an epicardial breakthrough. The SPB is a relatively lesser-known anatomical structure within the heart, comprising specialized myocardial fibers located between the septum and the pulmonary veins. While its precise function and significance in cardiac electrophysiology have not been extensively studied, emerging evidence suggests that abnormalities or alterations in the SPB may contribute to arrhythmogenic substrates such as atrial fibrillation and atrial flutter. The rationale for considering the involvement of SPB typically stems from several factors: (1) Anatomical proximity: The SPB is situated near critical structures involved in atrial conduction, such as the pulmonary veins and the atrial septum. Any anomalies or dysfunctions within the SPB could potentially disrupt normal conduction pathways, leading to arrhythmias. (2) Electrophysiological considerations: Studies have shown that the SPB contains specialized myocardial fibers with unique electrophysiological properties. Changes in the conduction velocity or refractoriness of these fibers may influence the overall atrial electrophysiology and contribute to arrhythmia maintenance. (3) Clinical observations: Clinical observations of patients with arrhythmias, particularly atrial fibrillation and atrial flutter, may reveal patterns or characteristics that suggest the involvement of the SPB. For example, patients who exhibit atypical arrhythmia presentations or who are refractory to conventional ablation techniques may prompt investigation into alternative arrhythmogenic substrates such as the SPB. The article provides a detailed description of the activation/tuning maps and ablation techniques employed in each case.

## Case presentation

Consistent with institutional guidelines, we secured patient consent before proceeding. All procedures were conducted under conscious sedation. A decapolar catheter from Boston Scientific Corporation (Marlborough, MA, USA) was strategically placed in the Coronary Sinus (CS). For mapping, we employed the Advisor™ FL mapping catheter (Abbott Park, Illinois, USA) in integrated with the Ensite Precision™ Mapping system from St Jude Medical (St. Paul, MN, USA) or the Pentaray catheter Biosense Webster Irvine, CA) integrated with the CARTO 3 system (Diamond Bar, CA, USA). Access to the left atrium (LA) was facilitated through dual transseptal punctures, assisted by long sheaths: an 8.5 French long sheath and a steerable sheath. Ablation was executed using a 3.5 mm-tip force-sensing irrigated ablation catheter, choosing between the SmartTouch SF from Biosense Webster and the TactiCath Sensor Enabled from Abbott.

### Case 1

**Fig. 1:**
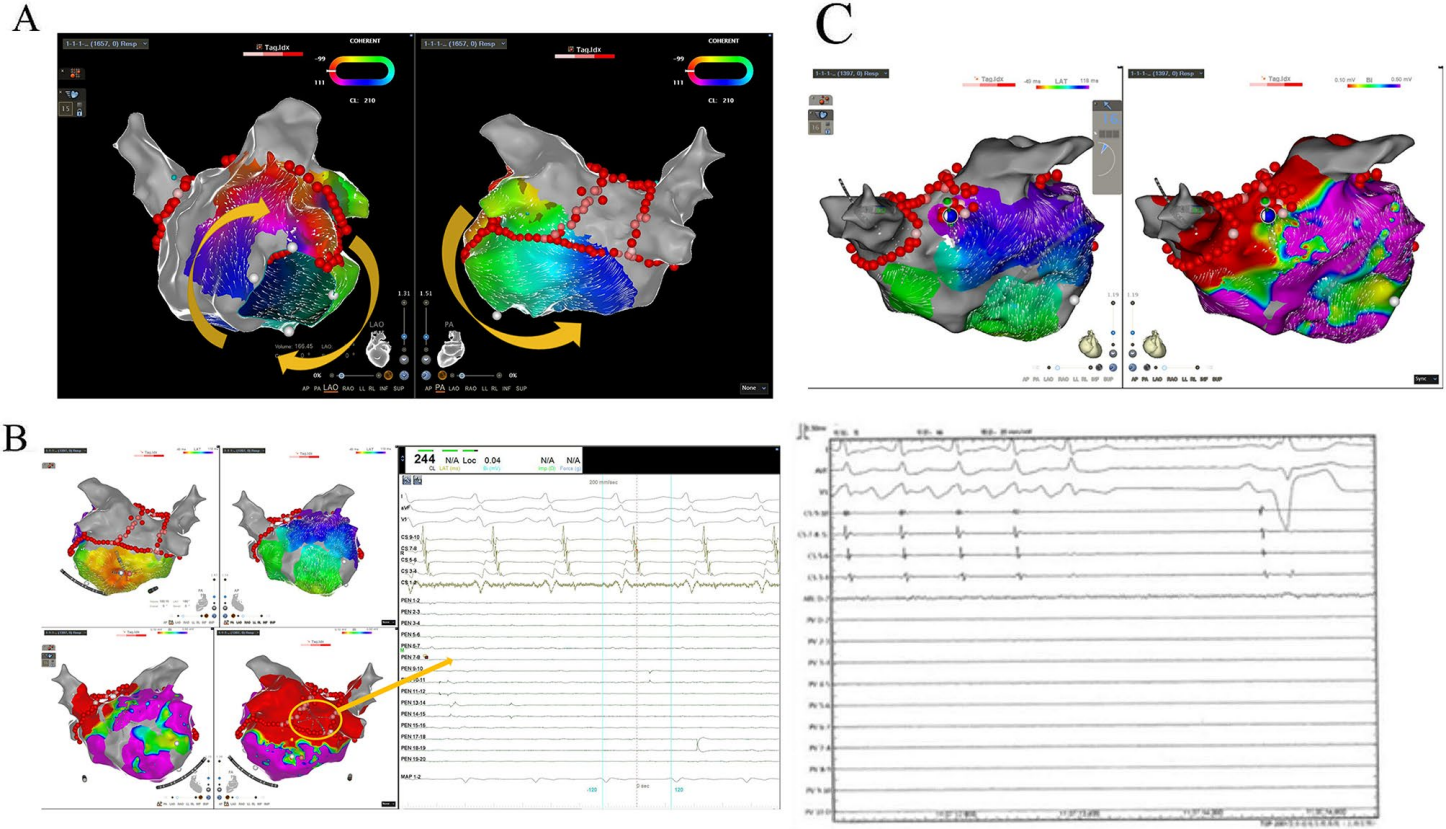
**A** Activation mapping in LA revealed a clockwise macroreentry around the mitral annulus; **B** Activation mapping revealed a CS activation sequence with the proximal taking precedence over the distal, exhibiting a 244 ms cycle length. The endocardial box region is blocked and the earliest activation site is located in the posterior wall below the floor line; **C** AFL was terminated during ablation at the anterior wall (slightly ahead of the roof)

**Fig. 2:**
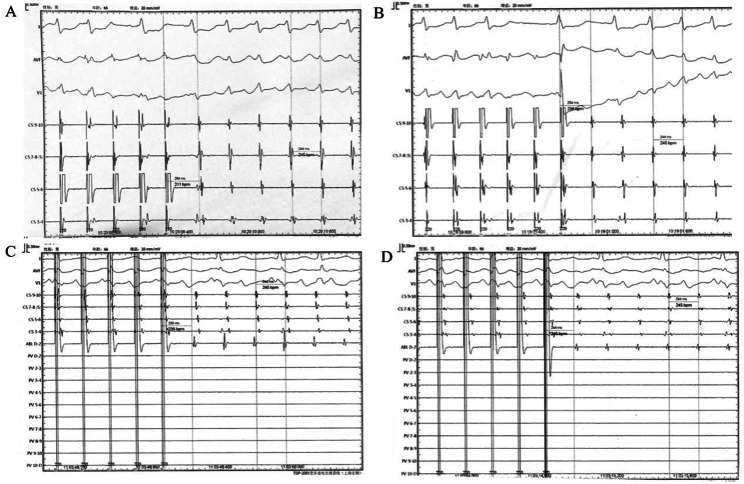
Synchronous pacing was observed at CS 5–6 (**A**) and CS 9–10 (**B**). The differences between the post-pacing interval (PPI) and the rapid heart rate cycle length (TCL) were 40 milliseconds and 10 milliseconds, respectively, indicating a higher likelihood of CS 9–10 site involvement in the reentrant circuit. The ablation catheter was positioned at the posterior wall (**C**) and roof (**D**) of the left atrium, and synchronous pacing was performed at each location. At the posterior wall location, the difference between PPI and TCL was 11 milliseconds, while at the roof location, it was 0 milliseconds

A 66-year-old male was evaluated for a nine-month history of chest discomfort attributed to atrial fibrillation. A “2C3L” ablation strategy was employed using the Smart Touch Surrounded Flow Catheter (W, control with a cut-off at 43 °C), involving PV isolation followed by linear ablations of the roof, the BOX ablation with a lower posterior line and mitral isthmus. After ablating the cava-tricuspid isthmus., the patient’s rhythm transitioned from atrial fibrillation to atrial flutter, characterized by a 210 ms cycle length and a CS activation sequence indicating precedence distal over proximal. Comprehensive left atrial mapping was executed using the Pentaray catheter, which revealed a clockwise macroreentry around the mitral annulus (Fig. 1A). While ablating in the distal coronary sinus, the rhythm transitioned to an alternative form of atrial flutter, with the coronary sinus activation sequence exhibiting a slightly preceding pattern over a cycle length of 244 ms (Fig. 1B). The endocardial box region is blocked and the earliest activation site is in the posterior wall below the floor line. 9-1028454the CS9-10 site is more likely in the of considering the CS catheter position (Fig. 2A-B). Furthermore, enhanced PPIs (255ms and 244ms) were achieved by pacing the ablation catheter at the posterior wall and roof of the left atrium (Fig. 2C-D). Considering the left atrial propagation, roof-dependent macro reentry without direct endocardial conduction became a plausible diagnosis. Typically, linear ablation at the posterior wall would be the intervention of choice to disrupt the circuit. However, given the pre-existing blockage in this patient’s posterior wall, an alternative strategy was pursued. We initially attempted to ablate the earliest activation site on the posterior wall, thinking it was the endocardial breakthrough site, but no effect was achieved. Subsequently, we performed anterior wall ablation, parallel and anterior to the roof line (50 W, targeting 550 Ablation Index), successfully terminating the flutter within 7 s (Fig. 1C). After a 30-minute observation period, no AF or AFL was induced using burst atrial pacing down to 180ms.

## Case 2

**Fig. 3:**
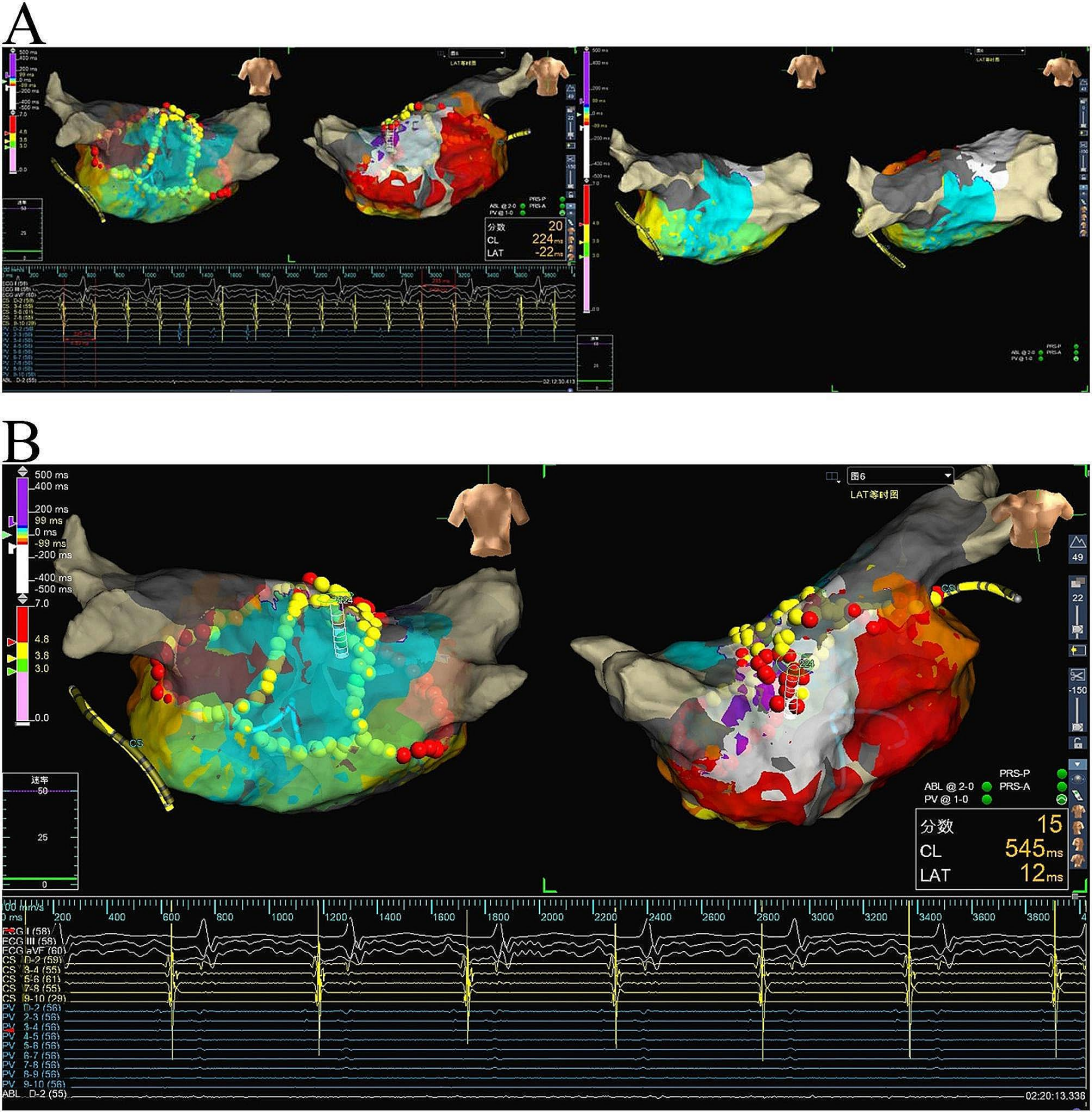
**A** Left: The tachycardia exhibited a cycle length of 220 ms, with the distal signal preceding the proximal one; Right: The endocardium in the roof has beed blocked; **B** Tachycardia was not induced during pacing under conditions with isoprenaline administration

A 78-year-old male with recurrent palpitations due to persistent atrial fibrillation underwent a procedure involving Pulmonary Vein Isolation (PVI) and Box Isolation. A power output of 45 W and targeting LSI of 4.0 was applied to the posterior left atrial wall and LSI of 5.0 at other left atrial sites. The irrigation rate varied between 25 and 30 ml/min. Following the PVI and Box ablation, an AFL with a cycle length (CL) of 220 ms emerged, where the distal coronary sinus signal preceded the proximal one (Fig. 3A). Activation mapping identified a macroreenry around the roof of the left atrium, it also showed a small portion of the CL absent in endocardial mapping, suggesting a potential epicardial component around the roof. Based on the experience of the previous case, we applied the third ablation line parallel and anterior to the roof line using 45 W (targeting LSI 4.0 posteriorly and LSI 5.0 anteriorly) with a contact force between 10 and 15 g. This action extended the CL from 220 ms to 235 ms immediately and effectively terminated the flutter in few seconds. Significantly, subsequent burst atrial pacing down to 200 ms did not induce any atrial fibrillation or flutter recurrence (Fig. 3B).

## Case 3

**Fig. 4:**
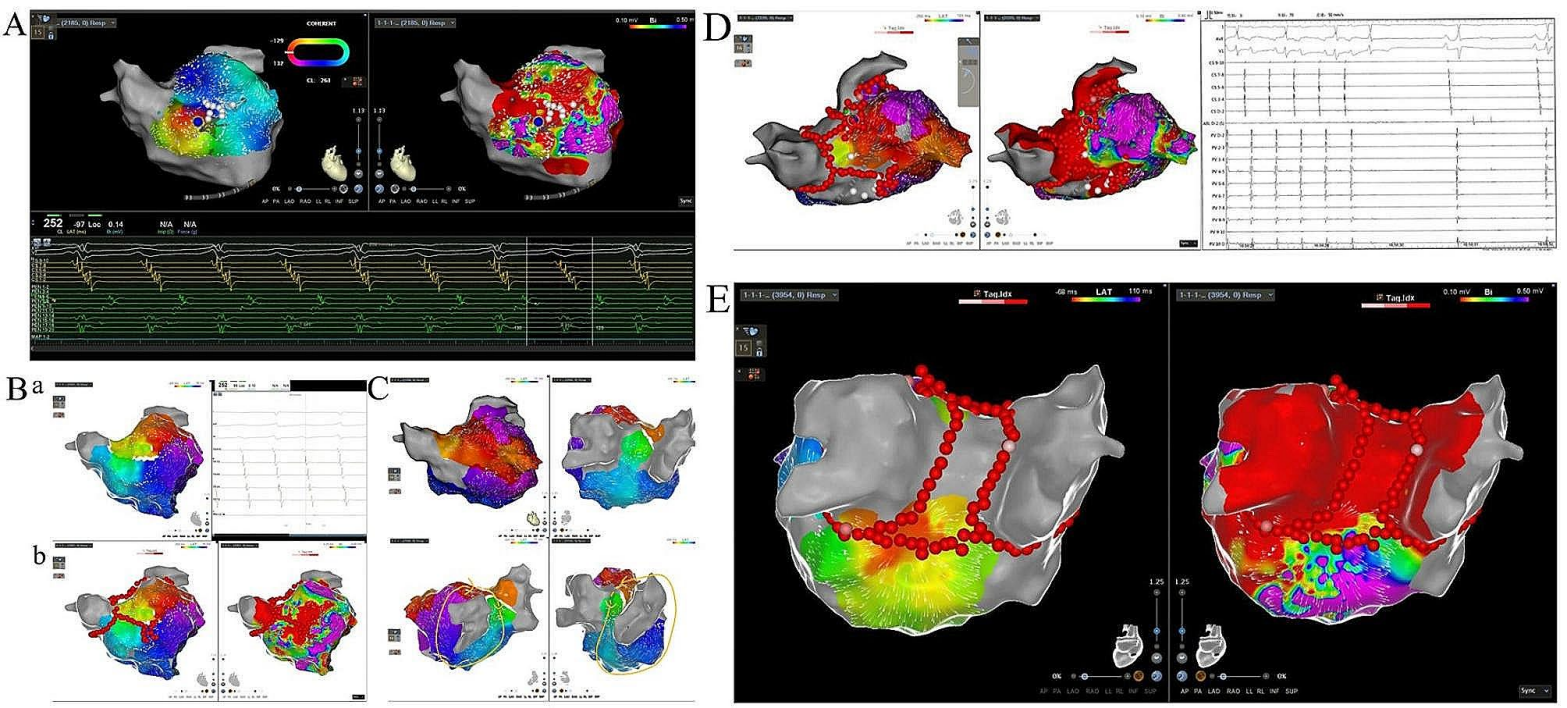
**A** The activation mapping of the left atrium reveals a region of low voltage at the anterior wall consisting of fragmented electrical potentials; **B** The activation mapping of the flutter, characterized by a stable cycle length of 252 ms, unveiled a reentry circuit localized at the anterior wall of the left atrium(a). A targeted isthmus ablation on the anterior wall successfully terminated the persistent flutter(b); **C** The activation map indicated the tachycardia as roof-dependent, with an endocardial block observed on the roof line, suggesting epicardial conduction through the septopulmonary bundle; **D** Tachycardia terminated while ablating at the anterior of the roof line within 10s; **E** The bottom-line ablation in the posterior wall was applied to achieve the box isolation

A 66-year-old female with a history of hypertension for one year presented with symptoms of chest tightness and shortness of breath for two years, was diagnosed with atrial fibrillation and flutter. The PV isolation ablation was performed firstly, then the tachycardia turned from atrial fibrillation into flutter with a varied cycle length (TCL), shifting between 250 ms and 290 ms. Activation mapping using Pentaray in the left atrium highlighted a low-voltage area on the anterior wall of the left atrium with fragmented potentials (Fig. 4A). To stabilize the TCL, we applied a modified ablation strategy focused on the low-voltage area and performed a linear ablation on the roof. This approach achieved TCL stabilization at a consistent 252 ms, with CS 9–10 predominating. Activation remapping revealed a reentry circuit on the anterior wall of the left atrium. A targeted isthmus ablation on the anterior wall successfully terminated the persistent flutter (Fig. 4B). Another flutter, with a cycle length of 363 ms and lead by CS 3–4, was subsequently induced during burst stimulation for validation. The activation map indicated the tachycardia as roof-dependent, with an endocardial block observed on the roof line, suggesting epicardial conduction through the SPB (Fig. 4C). A precise ablation, using a contact force above 10 g and a radiofrequency power of 50 W, was performed on the anterior part of the roof line. The flutter was terminated in 10 s (Fig. 4D). However, the flutter was induced again with a longer CL of 390ms and the same activation sequence. The bottom-line ablation in the posterior wall was applied to achieve the box isolation (Fig. 4E). AT was terminated during the final application and became no inducible.

Based on the three cases mentioned above, it can be observed that despite the ablation of the roof line, there is often still AFL involving roof-related reentry. The conventional approach involves performing focal or linear ablation at the earliest activation site below the roof line on the posterior wall of the left atrium. However, in many cases, even with posterior wall box isolation, AFL cannot be terminated. In such situations, consideration can be given to ablation in front of the roof line. Nevertheless, it should be noted that this approach is not always effective, and there are instances where posterior wall box isolation is still necessary.

## Discussion

The successful termination of atrial flutter (AFL) through catheter ablation at the anterior segment of the left atrial roof in all three cases represents a significant achievement and underscores the pivotal role of the SPB in AFL pathogenesis, particularly in cases dependent on the superior aspect of the atrium. This outcome highlights the clinical relevance of targeting the SPB during ablation procedures for AFL management. By demonstrating the effectiveness of ablating the SPB in terminating AFL, our study contributes to a deeper understanding of the anatomical and electrophysiological mechanisms underlying AFL. These findings provide valuable insights into potential therapeutic strategies aimed at modulating SPB function to prevent or manage AFL, ultimately improving patient outcomes in clinical practice.

The SPB spans the interatrial septum and envelops the left atrial appendage [7]. The SPB comprises an expansive set of subepicardial fibers originating from the interatrial groove beneath the Bachmann Bundle [8]. These fibers primarily ascend in a longitudinal direction and subsequently descend along the posterior wall between the orifices of the right and left pulmonary veins. The distinctive architecture of this bundle, coupled with the resulting changes in conduction anisotropy, constitutes the three-dimensional substrate implicated in atrial fibrillation and atrial flutter. It is hypothesized to play a pivotal role in atrial conduction. The SPB’s association with roof-dependent AFL could elucidate the unconventional presentations and reactions to standard treatments [9].

Achieving a transmural block of the roof line is challenging [7, 9]. Therapeutic strategies for roof-dependent atypical AFL frequently target the SPB, specifically focusing the ablation at the entrant point, which is primarily located on the posterior wall [10]. Recent studies noticed a disparity between the endocardial electrical activity (evidenced by absent or dissociated electrograms) and the epicardial activity (which sustains the atrial flutter). This electrical divergence between the endo- and epicardium might be attributed to the bilayer muscular structure of the left atrium [11]. Supplementing the original lines with additional ablation using higher power for extended durations has been suggested as an alternative approach. However, this method could also potentially increase the risk of esophageal injury. A recent study has reported a promising method that identifying and eliminating the endocardial breakthrough site of epicardial conduction on the anterior LA wall via the SPB, can assist in completing the PWI and terminating the flutter [12].

In the three cases presented, the SPB proved pivotal in the pathogenesis of roof-dependent atrial flutter. The unique electrocardiographic patterns displayed by these patients, along with their response to catheter ablation, underscored the suspected involvement of the SPB. The consistent cessation of the arrhythmia through catheter ablation at the anterior segment of the left atrial roof in all three cases further bolsters the supposition of the SPB’s role. In our first two cases, ablation was unsuccessful despite clear posterior wall isolation; however, opting for anterior roof line ablation unexpectedly proved to be successful. Two possible reasons might explain why the posterior wall isolation fails while the anterior roof line ablation succeed. First, the SPB in the anterior wall might be closer to the endocardium, facilitating easier disruption by the transmural lesion. Second, there might be a willingness to apply higher power and force during ablation of the anterior wall compared to the posterior wall considering the ablation safety, especially to minimize damage to adjacent tissues. For the third patient, initial ablation of the anterior roof line terminated the AFL but did not yield complete success, necessitating the subsequent isolation ablation of the posterior wall. This emphasizes that an anterior roof line ablation strategy is not invariably effective. Both posterior wall box isolation and anterior roof line ablation appear to have their own unique advantages.

Our findings regarding the successful termination of atrial flutter (AFL) through catheter ablation at the anterior segment of the left atrial roof corroborate and extend existing literature on the role of the SPB in AFL pathogenesis. Several prior studies have investigated the efficacy of SPB ablation in managing AFL, albeit with variations in patient populations, ablation techniques, and procedural outcomes. For instance, Liang et al. [13]. demonstrated a similar success rate in AFL termination through SPB ablation, albeit in a smaller cohort of patients with AFL refractory to conventional ablation strategies. At the same time, Yoshida et al. [14]. reported Epicardial conduction across the roof line is common and requires careful electrogram analysis to detect. In such cases, a floor line can be an effective alternative strategy, with clear validation criteria.

Our study builds upon these findings by presenting a larger cohort of patients and achieving consistent success in AFL termination through SPB ablation. Furthermore, by focusing specifically on AFL cases dependent on the superior aspect of the atrium, we provide valuable insights into the anatomical and electrophysiological characteristics of SPB involvement in this subset of AFL patients.

To the best of our knowledge, this is the first report to describe this observation. This innovative tactic offers a fresh therapeutic approach for atypical AFL that’s roof-independent and remains resistant to posterior wall and roof line isolation ablations.

It’s crucial to underscore that, although these findings appear promising, they stem from a limited set of cases. Further and more researches are needed to validate these findings and to explore the potential therapeutic implications.

## Limitation

This case report has several limitations that should be considered when interpreting the findings. Firstly, the study is based on a small sample size, including only three significant clinical cases. The limited number of cases may restrict the generalizability of the findings to a broader population. Future studies with larger sample sizes are needed to validate the involvement of SPB in these specific cardiac arrhythmias. Secondly, there may be a potential selection bias in the case selection process. The cases were chosen based on their unique electrocardiographic patterns and positive response to catheter ablation. This selection process may not accurately represent the entire population of patients with similar arrhythmias. Future studies should consider a more systematic and unbiased approach to case selection. Thirdly, the absence of a control group in this case report limits the ability to establish a causal relationship between the involvement of SPB and the observed arrhythmias. Including a control group in future research would allow for better evaluation of the specific contribution of SPB in these cardiac arrhythmias. Furthermore, the generalizability of the findings may be limited. Variations in patient characteristics, underlying conditions, and other factors may influence the involvement of SPB in different populations. Therefore, caution should be exercised when extrapolating these findings to other patient groups. Additionally, the lack of long-term follow-up data in this case report is another limitation. The focus of this report was primarily on the acute response to catheter ablation in terminating the arrhythmias. Long-term follow-up data on the recurrence rates and overall outcomes of the patients were not included. Future studies should incorporate long-term follow-up to assess the durability of the ablation procedure and the long-term prognosis for patients with SPB involvement. These limitations highlight the need for further research with larger sample sizes, rigorous study designs, and long-term follow-up to confirm the role of SPB in these specific cardiac arrhythmias and establish its clinical significance.

## Conclusion

The successful termination of AFL by catheter ablation at the anterior segment of the left atrial roof in all three cases provides compelling evidence for the crucial role of the SPB in the pathogenesis of AFL dependent on the superior aspect of the atrium.

## Data Availability

All data generated or analyzed during this study are included in this published article.
